# Research on Attitude Models and Antenna Phase Center Correction for Jason-3 Satellite Orbit Determination

**DOI:** 10.3390/s19102408

**Published:** 2019-05-27

**Authors:** Mingming Liu, Yunbin Yuan, Jikun Ou, Yanju Chai

**Affiliations:** 1 State Key Laboratory of Geodesy and Earth’s Dynamics, Institute of Geodesy and Geophysics, Chinese Academy of Sciences, No. 340 Xudong Rd., Wuhan 430077, China; ojk@asch.whigg.ac.cn (J.O.); cyjigg@asch.whigg.ac.cn (Y.C.); 2 University of Chinese Academy of Sciences, No. 19A Yuquan Road, Beijing 100049, China

**Keywords:** Jason-3 satellite, attitude modes, receiver phase center offset and variation (PCO/PCV), spaceborne GPS, precise orbit determination (POD), accuracy validation

## Abstract

We focused on the researches of two models used for Jason-3 precise orbit determination (POD)—Jason-3 attitude modes and receiver phase center variation (PCV) model. A combined attitude mode for the Jason-3 satellite is designed based on experimental analysis used in some special cases, such as in the absence of quaternions or when inconvenient to use. We researched the linking of satellite attitude with antenna phase center. Specially, to verify the validity of the combined attitude, we analyzed the effects of different attitude modes on receiver phase center offset (PCO) estimation, PCO correction and POD. Meanwhile, the difference analysis of PCO correction based on attitude modes also contains the combined attitude modeling processes. The POD results showed that the orbital accuracies with the combined attitude are slightly more stable than those with attitude event file. By introducing receiver PCVs into POD, the mean residuals root-mean-square (RMS) is reduced by 1.9 mm and orbital 3D-RMS position difference is improved by 5.7 mm. The eight schemes were designed to integratedly verify the effectiveness of different attitude modes and receiver PCVs model. The results conclude that the accuracy using the combined attitude is higher than that of event file, which also prove the feasibility of the combined attitude in integrated POD and it can be as a revision of attitude event file. Using all mentioned attitude modes, the orbital accuracy by introducing PCVs can be improved by the millimeter level. The integrated effects of attitude modes and receiver PCVs on POD are almost consistent with the effects of a single variable. The optimal results of Jason-3 POD indicate that orbital mean radial RMS is close to 1 cm, and the 3D-RMS position difference is within 3 cm.

## 1. Introduction

Jason-3 is a high-accuracy ocean altimeter satellite, successfully launched on 17 January 2016, which is a follow-on mission to OSTM/Jason-2. The Jason-3 satellite orbit is non-Sun-synchronous, 1336 km (2 h per revolution around the Earth) with a 66.038° inclination and an orbital repeat cycle of 9.9 days [1]. Investigators aim to produce a time series of orbits for all altimeter satellite missions (TOPEX/Poseidon, Jason-1 and Jason-2) that are consistent in their geophysical models, reference frames and measurement model corrections [2], supporting predictions of extreme weather conditions, oceanography and climate changes. The LEO satellite orbits can be obtained with the centimeter-level accuracy by processing GPS-only observation data [3,4]. Reduced-dynamic POD method was proposed by Wu [5] using velocity or acceleration pulses to adjust the weights of geometry observations and orbital dynamic models [5,6,7]. The precise GPS receiver was equipped into Jason-3 satellite [1,8]; in this way, the accuracy of GPS measurements can be fully exploited with the robustness offered by the orbit dynamic model [9].

Some scholars have researched the navigation satellites attitude [10,11,12,13] and the impacts on solar radiation pressure [14]. The researches on LEO satellite attitude modes and its impacts are limited [15,16,17], which can provide the technical reference for LEO satellites. Sometimes the measured attitude data in the form of quaternions are inconvenient to use or unable to know the changes of satellite attitude. As an alternative, investigators use the attitude event file to acquire attitude information. The above both data have update delay; attitude stability of event file is less than quaternions. The high-accuracy satellite orbits require the attitude to satisfy a certain stability in some cases, such as in the absence of quaternions or inconvenient to use. Thus, we designed a combined attitude model as an alternative or revision of attitude event file. It can also help us to perform some researches related to satellite attitude, such as solar radiation pressure, antenna phase center calibration and so on.

The terrestrial users can now benefit from the refined phase center modeling from some researches [18,19,20]; but receiver antenna phase patterns are unavailable for many space missions. The position of antenna phase center for spaceborne receiver often change due to systematical effects of the complex space environments, such as the near-field multipath [21,22]. Many scholars have modeled receiver phase center variation (PCV) for different LEO missions, such as GRACE [22,23], GOCE [24], TerraSAR-X [21], Jason-1/2 [3,16], Swarm [25], HY-2A [26], ZY-3 [27] and so on, and the orbital accuracies have been successfully improved. Therefore, research on the receiver PCVs provides a valid way to further improve the orbital accuracy and stability.

The satellite attitude affects orbits accuracy by affecting geometric calibration, especially antenna phase center offset (PCO), yet related research is rarely mentioned and analyzed. The antenna PCO and PCVs constitute the antenna phase center correction, which are highly correlated and coupled. Thus, a direct or indirect relationship exists among satellite attitude, PCO and PCVs. We tried to link satellite attitude with antenna phase center, and attempted to analyze their correlation. Some authoritative organizations have studied Jason-2 precise orbit determination (POD) [2,16,28,29], but so far there are almost no papers that involve the Jason-3 POD. Thus, we considers the Jason-3 satellite as an example.

The research contents include the different attitude modes and antenna phase center model, verifying the availability of the designed combined attitude and receiver PCVs model, and perform the POD to obtain stable orbits for Jason-3 satellite. Section 2 gives models for the combined attitude, antenna PCO/PCV and POD strategies. Section 3 discusses the experiments of attitude modes (related to receiver PCO) and receiver PCVs model, and their respective impacts on the POD. Section 4 introduces the above models into Jason-3 POD to integratedly compare and analyze the results. Finally, we summarize the conclusions.

## 2. Model for Attitude, Antenna PCO/PCV and Its POD Strategies

This section elaborates the models for Jason-3 attitude, receiver PCO/PCVs and POD strategies. We designed a combined attitude model for Jason-3, and clarified the model of receiver PCO estimation and its correction involving attitude; the receiver PCVs is modeled and described. The models and strategies of POD, including attitude information models and receiver PCO/PCV modeling, are given in this section.

### 2.1. Jason-3 Satellite Attitude

Satellite attitude refers to the orientation of the satellite body system (SBS) in a reference frame, specifically, the coordinate transformation matrix between the SBS and Earth-fixed system or inertial system [12,30]. The satellite attitude variation can affect non-gravitational perturbation by the changes of satellite force surface, and influence the geometric correction in the POD, specifically the antenna phase center. Large errors in satellite attitude would cause incorrect PCO estimation. Even if the PCO values are accurate, the inaccurate attitude can also cause PCO correction errors, which further generate incorrect orbits.

#### 2.1.1. Jason-3 Attitude Model

In general, the satellite attitude modes include nominal attitude and yaw-steering mode. For Jason satellites, in order to optimize the energy collected on the solar panel, ideal yaw steering should always be sinusoidal, but when beta is low that mean that the satellite would rotate back and forth very fast, exceeding the capacity of the attitude control; the sub-optimal fixed yaw attitude is used. Therefore, the Jason satellite attitude is a mixed mode of fixed yaw and yaw steering. Users who do not want to use quaternions should use an attitude model. The targeted attitude of the Jason-1/2 satellite [16] can be summarized as follows: When beta is higher, the platform is steered around the yaw direction to keep the *y* axis of SBS perpendicular to the Sun vector, which adopts yaw-steering mode. When beta is low, the yaw angle is fixed and the *x* axis is kept parallel to the along-track direction, which is nominal mode (in the form of fixed yaw used in more LEOs), but this targeted attitude is invariant due to its fixed threshold.

In the absence of quaternions during the POD, even the near-real-time OD, to obtain attitude information easily, we designed a combined attitude based on experimental analysis as an alternative or revision scheme. The relative formula for the yaw angle is expressed as:

(1) $$\varphi \;=\;\{\begin{array}{c} \frac{\pi }{2}\;+\;(\;\frac{\pi }{2}\;-\;\beta )\cdot \sin\mu ,where\;\beta >\beta_{0}\\ \;0\;,where-\beta_{0}\leq \beta \leq \beta_{0}\\ -\frac{\pi }{2}\;-\;(\;\frac{\pi }{2}\;+\;\beta \;)\cdot \sin\mu ,where\;\beta <-\beta_{0} \end{array}$$

where φ denotes the yaw angle, that is, the angle between the *x* axis of the SBS and the velocity direction; β is the angle between the Sun vector and the satellite orbit plane; $\beta_{0}$ is the threshold of the attitude transition between yaw steering and fixed yaw; and μ is the orbit angle, that is, the geocentric angle between the spacecraft position vector and the orbit midnight, growing with the satellite’s motion [30]. The calculations of μ and β are provided in [10,30] and will not be described here.

In this method, we dealt with some details correspondingly. The acquisition of transition threshold is based on the difference between quaternions and attitude model. When the difference between fixed model and quaternions exceeds a certain value (such as 1 cm), the beta corresponding to this value is considered as the transition threshold of combined model. By introducing a transition threshold into Equation (1) one may get attitude information. The differences between the combined attitude and attitude event file essentially are the difference of attitude transition nodes. Even if the quaternions have update delays, the combined model can maintain the attitude information for a period of time. Similar to an event file, the threshold of combined model varies for different periods and represents the short-term results. For example, the value 14.6° is adopted based on the experimental analysis of PCO correction for a certain period of time data in Section 3.1.2. In addition, when β is close to a certain value (such as 1.06°), the Jason-3 satellite is flipped around the yaw axis (the *z* axis of SBS); the attitudes would be correspondingly dealt with using a rotation matrix. In turn, the transition threshold of combined attitude can provide the basis of data selection for PCO estimation or other researches related to satellite attitude. Based on the combined attitude, attitude information is available easily without event file, which can be applied as a revision of event file to get stable attitude information and orbits.

#### 2.1.2. Models of PCO Estimation and PCO Correction

**Figure 1:**
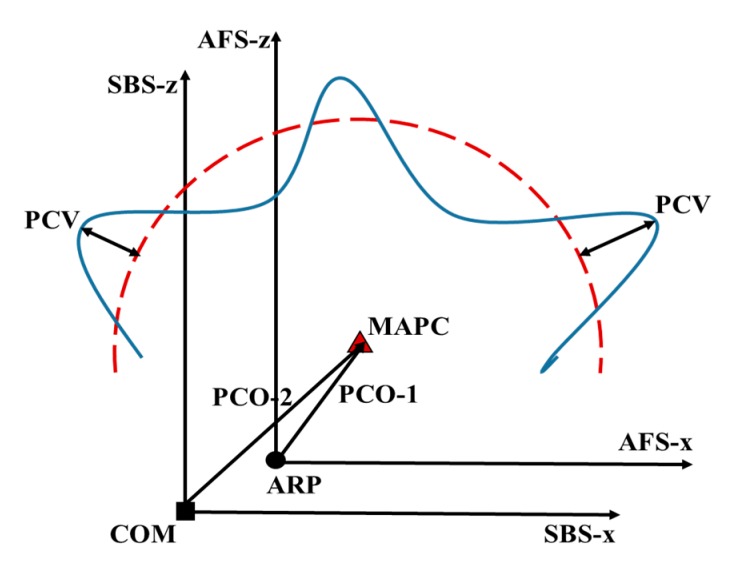
Antenna PCO and PCV model.

In general, the differences between the locations of antenna phase center and antenna reference points (ARP) are referred to as the phase center correction [22,24]; it consists of the PCO vector and PCVs. The antenna PCO can be defined as the vector from the ARP to the mean antenna phase center (MAPC) in the antenna-fixed system, and can also be described as the vector from center of mass (COM) to mean antenna phase center in the SBS. The difference between the two definitions is a vector of the ARP with respect to the COM. The second definition is applied in the researches. The coordinates of a priori PCO are generally provided by ground calibrations [21,31]. The actual PCO differs from the priori PCO due to ground calibration error, fuel consumption, and deformation caused by in-flight temperature variations [31]. The fuel reduction of the satellite’s tank would cause offsets of different magnitudes of the satellite COM. The regular updates of the receiver PCO coordinates are indispensable to ensure high-accuracy orbits. In addition, the schematic diagram of antenna PCO and PCV model is shown as Figure 1.

The receiver PCO as the unknown parameter is usually introduced into observation equations of the POD. The observation equation involving LEO satellite attitude can be represented as:

(2) $$L=A\cdot \vec{x}\;+\;B\cdot {\vec{e}}^{T}\cdot ATT_{S}^{I}\cdot \vec{PCO}\;+\;C\cdot dtr+D\cdot \vec{n}+\varepsilon_{L}$$

where *L* denotes the phase observation of the ionosphere-free combination; $\vec{x}$ denotes the parameter vectors of the initial orbits and empirical acceleration; $\vec{PCO}$ denotes the receiver PCO vector in the SBS or antenna-fixed system; $\vec{e}$ denotes the unit vector of the incident direction of navigation satellite signal; $ATT_{S}^{I}$ denotes the LEO attitude; dtr denotes the receiver clock error; $\vec{n}$ denotes the ambiguity parameter; A, B, C, D is the corresponding coefficient matrix; $\varepsilon_{L}$ denotes the phase observation noise.

In general, for fixed-yaw satellites, the x- and y-components of PCO estimation are unreliable and unstable [31,32]; only the z-direction results of PCO estimation are feasible. Considering the mixed attitude and slightly canted antenna for Jason-3 satellite, the yaw-steering data were selected to estimate the z-direction results of PCO vector. The thresholds of attitude transition would be used to select suitable data.

The formula of the PCO correction in the inertial system is given by:

(3) $$\vec{\Delta PCO}=ATT_{S}^{I}\cdot \vec{PCO}$$

where $ATT_{S}^{I}$ denotes the satellite attitude; $\vec{PCO}$ denotes the PCO vector in the SBS; and $\vec{\Delta PCO}$ denotes the PCO correction in the inertial system. The difference in PCO correction based on different attitude modes can provide a defined method for the transition threshold of the combined attitude; it is also a validation way for attitude modes.

### 2.2. Jason-3 Receiver PCVs

When the orbital accuracy is close to centimeter level, the in-flight bias of receiver PCVs would become main effect on POD, and neglecting receiver PCVs may introduce systematic errors [21,22]. The receiver PCVs depend on the signal frequency and receiving direction; it is modeled by the distance between the instantaneous phase center and the mean phase center. The errors of the signal range caused by receiver PCO and PCVs are generally expressed by:

(4) $$\rho_{ARP}=\;\vec{PCO}\cdot \vec{e}(\alpha ,\beta )+PCV(\alpha ,\beta )$$

where $\vec{e}$ denotes the unit vector of the satellite signal incident direction; $\vec{PCO}$ and PCV denotes the receiver PCO vector and PCVs; α,β are the azimuth and elevation angle of the signal propagative direction.

The PCV estimation methods usually include the direct and the residual approach [22,26]. The direct approach considers receiver PCVs and orbital parameters together as unknown parameters in the POD. This approach depends on parameter information and computer storage. Jäggi [22] indicated that the direct approach may introduce systematic errors but the reason remains unknown. The residual approach, as an extensively employed method for in-flight calibration of receiver PCVs, was applied to the Jason-1 satellite by Haines and the orbital accuracy was successfully improved [3]. This approach models the residuals derived from POD according to the azimuth and zenith to divide the whole antenna space into grids, and the residual of each grid point is calculated by averaging the surrounding residuals. The residual approach substantially models PCVs according to the accidental error characteristics, and the results are relatively stable. There is no direct relationship between the obtained PCVs and attitude model. The phase residuals model of the current period can be adopted to correct for PCVs of the previous, current, and future periods, because receiver PCVs from different periods has similar systematic characteristics [23]. The research on receiver PCVs modeling of Jason-3 is lacking; thus, we modeled receiver PCVs and studied whether it can improve Jason-3 orbital accuracy.

### 2.3. Jason-3 POD Models and Strategies

For new models or methods used in POD, including the combined attitude model, the most effective and practical evaluation method is to evaluate the accuracy performance of orbits obtained from them. We took the POD for example to analyze and validate the above models. The LEO POD usually adopts an undifferenced reduced-dynamic method to eliminate the error of ionospheric first-order term by forming the dual-frequency ionosphere-free combination [21,33,34]. The settings of model correction and parameters for perturbative force, parameter estimation and strategies in the POD are shown in Table 1.

Currently, there are no reference paper about the precise orbits of Jason-3 satellite to show its orbital precision. But, the Jason-3 Products Handbook [1] (Page 42, Section 5.1. Precise Orbits) mentioned that “CNES (the French Space Research Center) has the responsibility for producing the orbit ephemerides for the Jason-3 data products. The Jason-3 OGDRs provide a navigator orbit that has radial accuracies better than 5 cm (RMS), the Jason-3 IGDRs provide a preliminary orbit that has radial accuracies better than 2.5 cm (RMS), while the GDRs provide a precise orbit that has radial accuracies better than 1.5 cm (RMS).” In this paper, we use the GDR-E precise orbits (ftp://cddis.gsfc.nasa.gov/doris/products/orbits/ssa/ja3/GDR-E/) provided by CNES for Jason-3 as a reference. Thus, the radial accuracies of CNES reference orbits are better than 1.5 cm (RMS) according to the Jason-3 Products Handbook. Meanwhile, the POD strategies about CNES orbits in the above link. CNES orbits are obtained by joint orbit determination using GPS, DORIS and SLR technologies. In its POD strategies, the attitude model of CNES precise orbits applied quaternions and solar panel orientation from control center, completed by nominal yaw steering law when necessary. The GPS receiver PCO and PCV by CNES precise orbits are consistent with constellation orbits and clocks (IGS08 ANTEX) and pre-launch GPS receiver phase map, respectively. That is, for GPS receiver PCV of Jason-3, CNES GDR-E orbits did not in-flight adjust GPS receiver phase map; it is different from our strategies, which is based on in-flight residuals modeling. 

## 3. Experiments for Jason-3 Attitude Modes and Receiver PCVs Calibration

In this part, we study the linking of the satellite attitude with the antenna phase center, and we focus on the availability of the combined attitude mode and the effects of receiver PCVs on POD.

### 3.1. Effect of Jason-3 Attitude Modes on Receiver PCO and POD

To verify the validity of the combined attitude mode, the experiments analyzed the effect of different attitude modes on receiver PCO, including the PCO estimation and its correction, and the effect on POD by internal and external orbit validation. The normal (fixed-yaw) attitude mode was selected in tests because it is adopted in more LEO satellites and beneficial to the combined attitude modeling.

#### 3.1.1. Effect of Jason-3 Attitude Modes on PCO Estimation

The orbital time series of all altimeter satellites are required to be consistent in their geophysical models, reference frame and measurement model corrections, for which the corresponding parameters between Jason-2 and Jason-3 should have this inheritance. Thus, we considered the receiver PCO vector of Jason-2 as the initial PCO of the Jason-3 satellite, which the z-offset of PCO is −521.7 mm for the SBS. Given the particular geometry of the orbit and its complex interaction with the attitude, we estimated the z-offset of PCO using one beta cycle of data without fixed-yaw data about more than 90 days, according to the thresholds of attitude transition. Due to data reasons used, the results based on the combined attitude and event file are consistent with each other, only the nominal mode, combined mode and quaternions were selected to test in this section.

By POD experiments, we have determined that the z-direction of PCO estimation using quaternions based on an initial value of −521.7 mm is more accurate; thus, we considered the estimation results (−518.7 mm) as the reference value. The initial z-direction bias of receiver PCO with respect to the reference value are randomly increased to −24.4 mm and −100.2 mm; the z-offset of PCO in both cases were estimated based on three attitude modes. To compare the differences, the initial bias and estimated bias with respect to the reference value are listed in Table 2. The values in parenthesis indicate the percentage of the estimated PCO bias with respect to the initial bias.

In both cases of Table 2, the estimated results with nominal attitude (36.2 and 124.8 mm) are the worst; results with the combined attitude (−18.8 and −62.8 mm) are relatively closer to the reference value. These results indicate that different attitude modes have different impacts on PCO estimation. In tests, the estimated receiver PCO bias based on the nominal attitude are about 148.4% and 124.6% with respect to initial bias, those with combined model are about 77.0% and 62.7%, and those with quaternions are 14.3% and 5.1%. The receiver PCO does not need to be updated in real time and only needs to be revised regularly. In POD, we should use more accurate attitude model firstly. If the PCO needs to be updated, it will be estimated; if not need, it will not be estimated.

#### 3.1.2. Effect of Different Attitude Modes on PCO Correction (Modeling Analysis of the Combined Attitude)

The PCO correction error can directly make the calibration of satellite mass center inaccurate. Supposing that receiver PCO provided from a ground or an in-flight estimation is accurate, the differences in PCO correction are calculated using different attitude modes. The difference analysis can be as the validation way and the defined method for the transition nodes of the combined attitude mode.

**Figure 2:**
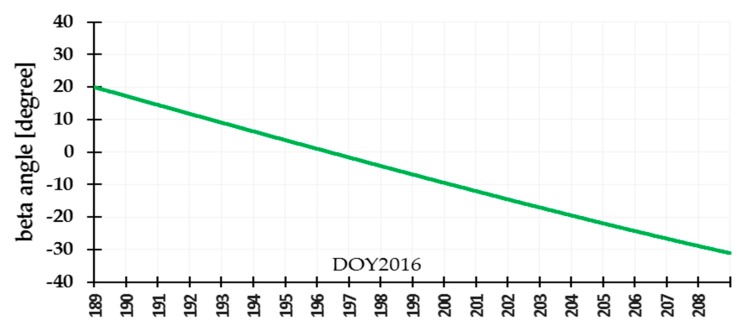
Beta angles variation for Jason-3 satellite.

**Figure 3:**
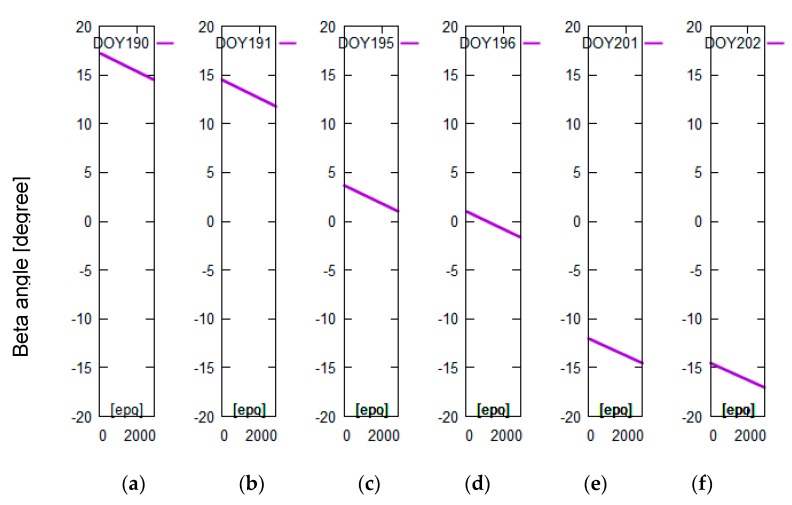
Variation of Jason-3 beta angles near ±14.6° and 1.06° (the transition threshold of the combined attitude) in DOY190-191 (**a**,**b**), DOY195-196 (**c**,**d**) and DOY201-202 (**e**,**f**).

**Figure 4:**
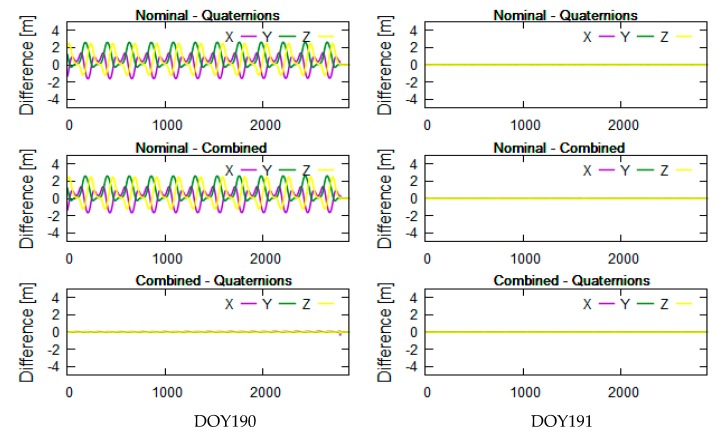
Differences in PCO correction using different attitude modes near +14.6°.

**Figure 5:**
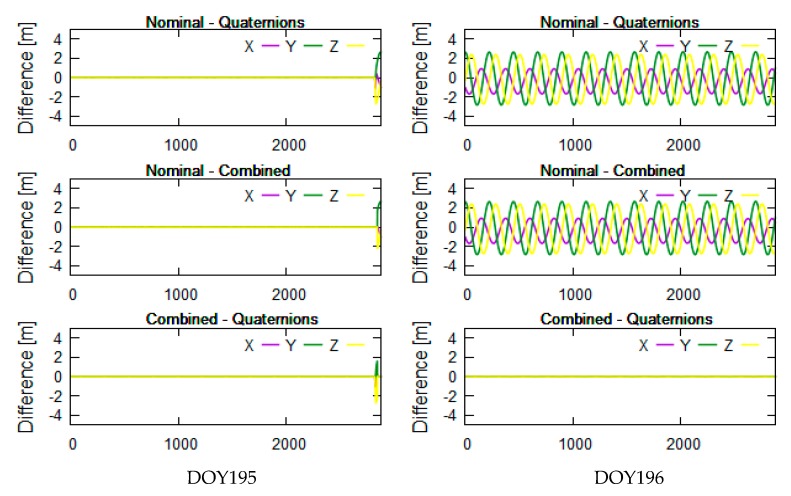
Differences in PCO correction using different attitude modes near 1.06°.

**Figure 6:**
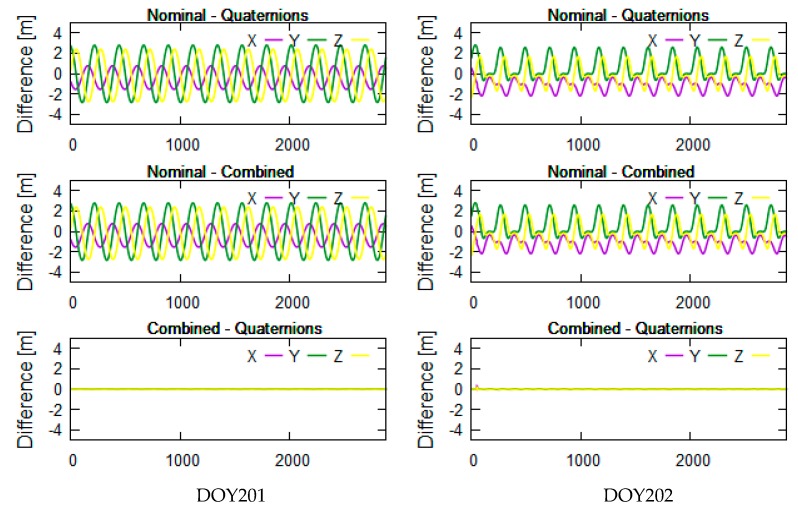
Differences in PCO correction using different attitude modes near −14.6°.

The yaw angle is mainly controlled by the beta angle of the satellite. To better analyze the correlation between beta angles and differences in PCO correction, we selected related data which the beta angles encompass the threshold of attitude transition. Considering the limitation of length of the paper, we presented the analysis results related to 20 days of data: days of year (DOY) 189-208 in 2016. The related beta angles were calculated and linearly vary from −35° to 20° in Figure 2. The tests didn’t selected one beta cycle of data because the transition threshold of the combined attitude has characteristics with short-term change with time, which is better for getting the transition threshold and POD. Figure 3a–f shows that the Jason-3 beta angles are nearly the transition threshold (±14.6° and 1.06° are obtained from test analysis in this section) of the combined attitude in DOY190-191 (Figure 3a,b), DOY195-196 (Figure 3c,d) and DOY201-202 (Figure 3e,f). In Figure 4, Figure 5 and Figure 6, the units of the *x*-axis are epoch of day.

The left and right sides of Figure 4, Figure 5 and Figure 6 reflect the differences in receiver PCO correction in the inertial system using different attitude modes in DOY190-191, DOY195-196, and DOY201-202, respectively. From top to bottom are the differences in PCO correction between nominal attitude and quaternions, between nominal and combined attitude, and between combined attitude and quaternions.

The Jason-3 beta angles are nearly +14.6° in DOY190-191, nearly 1.06° in DOY195-196 and nearly −14.6° in DOY201-202 (see Figure 3). In Figure 4, Figure 5 and Figure 6, the differences in PCO correction between nominal attitude and other attitude modes reached a maximum of 2 m in DOY190, DOY196 and DOY201-202. In DOY191 and DOY195, the differences between three attitude modes are almost 0, which indicates that three attitude modes are available in these cases. In DOY190-191, DOY196, DOY201-202, the results using combined attitude are almost consistent with quaternions; it shows that the combined attitude and quaternions can be employed in PCO correction. At the end of DOY195, some abnormal values are shown in Figure 5; we searched for the attitude event information and discovered a process about yaw flip of 10 min at 21 o’clock on that day. And all attitude models have the modeling error that caused the poor results.

In Figure 4, Figure 5 and Figure 6, the nominal attitude is not feasible and can cause the meter-level correction error. The results of PCO correction using the combined attitude are almost consistent with those using quaternions. In fact, the difference analysis in PCO correction between quaternions and nominal mode are also the basis of the combined attitude modeling in this section.

#### 3.1.3. Orbital Accuracy Validation with Different Attitude Modes

We compare and analyze the differences in orbital accuracy with different attitude modes, including the combined attitude, event file and quaternions. In the event file, the transition thresholds between sinusoidal and fixed modes (between yaw-steering and normal modes) are +14.4° and −14.8°, corresponding to DOY189-208. The thresholds in the combined attitude model are +14.6° and −14.6°, respectively. The event file must be used in conjunction with the model, so the differences between the combined attitude and event file essentially are the difference of transition thresholds between sinusoidal and fixed yaws, and yaw flip event.

**Figure 7:**
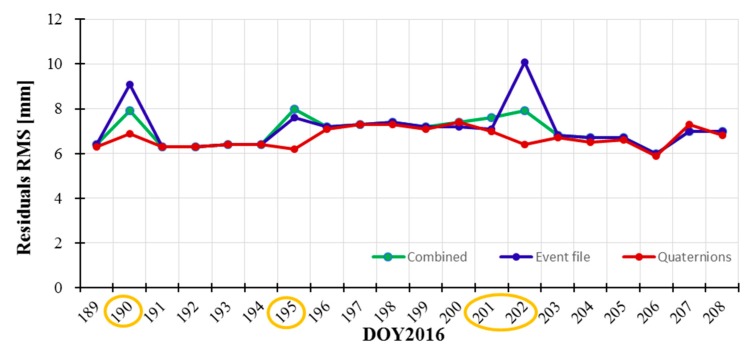
Residuals RMS based on different attitude modes.

**Figure 8:**
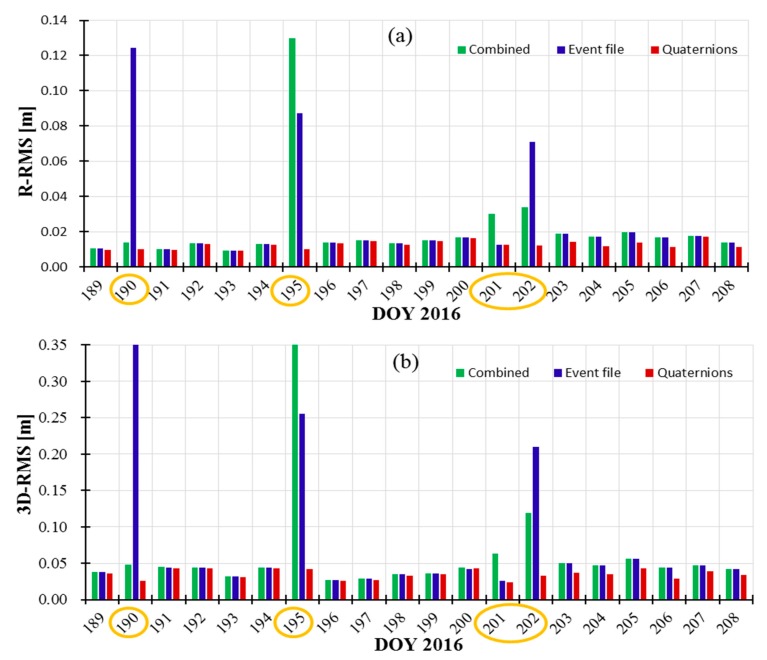
Comparison of orbital accuracies (RMS) on R direction (**a**) and 3D position (**b**) based on different attitude modes (using combined attitude in green, using event file in blue and using quaternions in red).

To compare the differences between the combined attitude, event file and quaternions, we also perform the tests on POD. The residuals RMS with different attitude modes are showed in Figure 7. Figure 8a,b denotes the accuracy comparisons in radial (R) directions and 3D position between our orbits and the CNES orbits. In Figure 7 and Figure 8, we marked the obvious differences with orange cycles on DOY190, DOY195, and DOY201-202. Here, noted that the attitude transition node between yaw-steering and nominal modes are in DOY190, DOY201-202; the attitude node of yaw flip are in DOY195, so on these days, the RMS variations of residuals, R direction and 3D position with the combined attitude and event file are larger than other days due to modeling errors.

In Figure 7, the residuals RMS based on the combined attitude (in green) is more stable than the results of event file (in blue); residual RMS with quaternions is least and most stable. In DOY190, DOY195 and DOY202, the orbital accuracies of R direction and 3D position with event file (in blue) are large than 6 cm and 20 cm, respectively, as shown in Figure 8. The accuracies with the combined attitude (in green) are larger than 12 cm and 35 cm in DOY195, respectively. Overall, the orbital accuracies of R direction and 3D position using the combined attitude (in green) are slightly more stable than those using event file (in blue). And the combined attitude model can be applied in Jason-3 POD as a revision of event file to get more stable attitude and orbits. We must emphasize that the combined attitude and event file are used to get attitude information based on the models, inevitably leading to modeling errors in attitude transition nodes (such as DOY195). The orbital accuracies with quaternions are best and most stable among three modes. In general, the orbital centimeter-level accuracies can be obtained using these three attitude modes.

### 3.2. Experiments of Receiver PCV Correction

**Figure 9:**
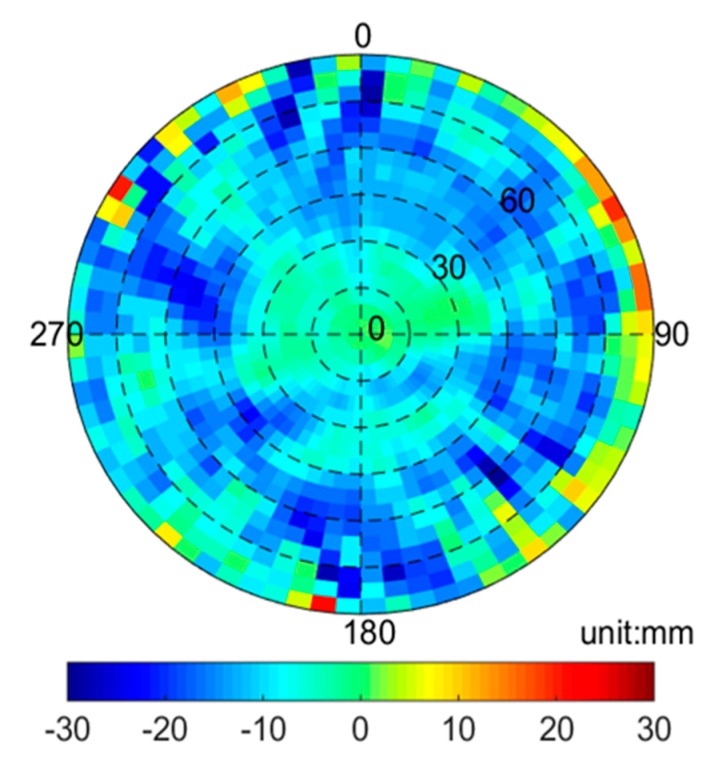
Receiver PCVs map from ionosphere-free combination for Jason-3 satellite.

The receiver PCVs of the Jason-3 satellite were modeled with the residual method. Since partial PCVs may be absorbed by receiver clock error, ambiguity and other parameters in the POD, iterative procedures are required to continuously correct PCVs, which means that the new results as increments are generated to add them to the last PCVs grid model with the same resolution in the form of 5° × 5° grids. This procedure was repeated several times to obtain a final receiver PCVs grid model of the Jason-3 satellite from ionosphere-free combinations. Considering the complex satellite attitude, we selected nine-month data to better model PCVs from April to December in 2016, including more than two full beta cycles of data. Figure 9 shows the receiver PCVs map of Jason-3 derived from ionosphere-free combination. The comparisons of orbit residuals, external orbits and SLR validation results were performed to reflect its impact on Jason-3 POD. 

#### 3.2.1. Residuals Comparison

**Figure 10:**
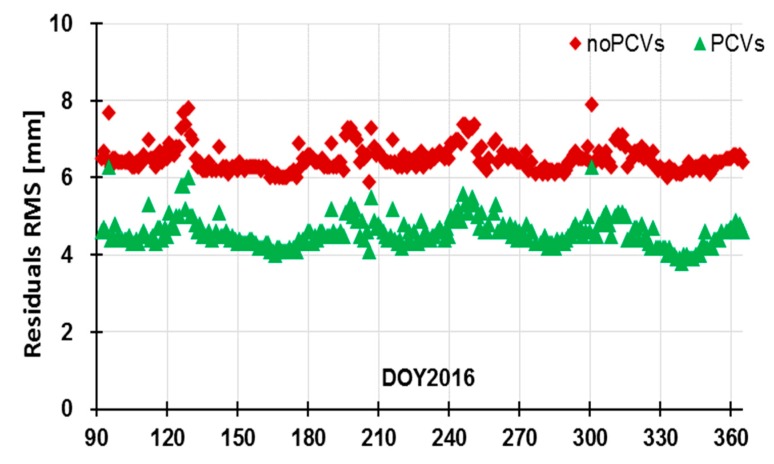
Residuals RMS statistics for Jason-3 POD.

In Figure 10, the residuals RMS of Jason-3 POD with and without PCVs map are approximately 4–6 and 6–8 mm, respectively. The mean residuals RMS with PCVs map are reduced from 6.5 to 4.6 mm; the internal accuracy is improved by 1.9 mm. These results are consistent with the noise level of the ionosphere-free combination of the carrier phase, which indicates that models and methods are reasonable for Jason-3 satellite.

#### 3.2.2. External Orbits Validation

The Jason-3 orbits provided by CNES were used as the reference orbits. Table 3 show the comparisons of the orbital external validation results with and without receiver PCVs in radial (R), along-track (T), cross-track (N) directions and 3D position. Compared with CNES orbits, the radial accuracies with and without PCVs are approximately 11.3 mm, which seems no obvious improvement. The accuracy improvements of 6 mm and 2 mm are obtained in the T and N direction; the mean 3D-RMS position difference has an improvement of approximately 5.7 mm. The accuracy of external validation by introducing PCVs map has the millimeter-level improvement.

#### 3.2.3. SLR Validation

Satellite Laser Ranging (SLR) validation is an important means to validate the orbital accuracy [32,36]. We have selected the SLR normal point data, corresponding with the periods of the Jason-3 POD (ftp://cddis.gsfc.nasa.gov). The SLR validation results denote the distance accuracy between the validated satellite and SLR station, which cannot validate the accuracies of the orbital position and components in each direction. The SLR validation are more exhibited as the radial accuracy when cut-off angle is set higher. Thus, the cut-off angles are respectively set to 0° and 60°, in which 0° validate errors of equivalent distance and 60° validate the orbital radial accuracy.

Table 4 lists the orbital SLR residuals statistics for Jason-3 satellite. When the cut-off angle is set to 60° and 0°, the residuals with PCVs are approximately −3.7 ± 13.2 mm and −0.8 ± 17.2 mm. The validated results with the cut-off angle of 60° and 0° show that the equivalent distance error by introducing PCVs is improved by 0.7 mm and 4.1 mm, respectively, compared with neglecting PCVs. The SLR validation results do not have the large systematic bias. The total results indicate that the orbital radial accuracies are approximately 1.4 cm, nearly 1 cm, and the equivalent distance errors are within 2 cm.

The above comparisons show that the orbital accuracies by introducing receiver PCVs map have the millimeter-level improvement. These results indicate that receiver PCVs should be considered for high-accuracy orbits, especially when the orbital accuracy is close to centimeter level.

## 4. Integrated Experiments

The above two strategies about attitude modes and PCV modeling are introduced into Jason-3 POD to integratedly compare their performance. The experiment selected 20 days data, the same as Section 3. Eight schemes for POD were designed: scheme 1 used nominal attitude without PCVs; scheme 2 used attitude event file without PCVs; scheme 3 used the combined model without PCVs; scheme 4 used quaternions data without PCVs; scheme 5 used nominal attitude with PCVs; scheme 6 used attitude event file with PCVs; scheme 7 used the combined model with PCVs; scheme 8 used quaternions data with PCVs. Limited by length of paper, we only utilized CNES orbits and SLR technology to validate the orbital accuracy for eight schemes.

Table 5 shows the validation accuracy of eight schemes using CNES orbits and SLR technology. The maximum errors with/without receiver PCVs with nominal attitude corresponds to the PCO correction errors (see Section 3.1.2). Compared with schemes 1 and 5, schemes 2 and 6, schemes 3 and 7, schemes 4 and 8, the 3D-RMS position difference by introducing receiver PCVs are improved by 13.8 mm, 2.6 mm, 3.7 mm, and 4.7 mm, and the distance errors with SLR validation are improved by 0.4 mm, 1.4 mm, 1.8 mm, and 1.1 mm, respectively. Compared with schemes 2 and 3, schemes 6 and 7, regardless of whether PCVs are introduced, the accuracy using the combined attitude is higher than that of attitude event file (in red marks), which also prove the feasibility of the combined attitude in integrated POD. The combined attitude can be as a revision of event file to get more stable attitude and orbits. By introducing receiver PCVs map, orbital accuracy can be improved by millimeter level based on the combined attitude, event file, and quaternions. The integrated effects of attitude modes and receiver PCVs on POD are almost consistent with the effect of a single variable. Among the eight schemes, the optimal POD results in the R, T and N directions and 3D-RMS position difference are approximately 1.33 cm, 2.38 cm, 1.17 cm, and 2.97 cm, respectively.

**Figure 11:**
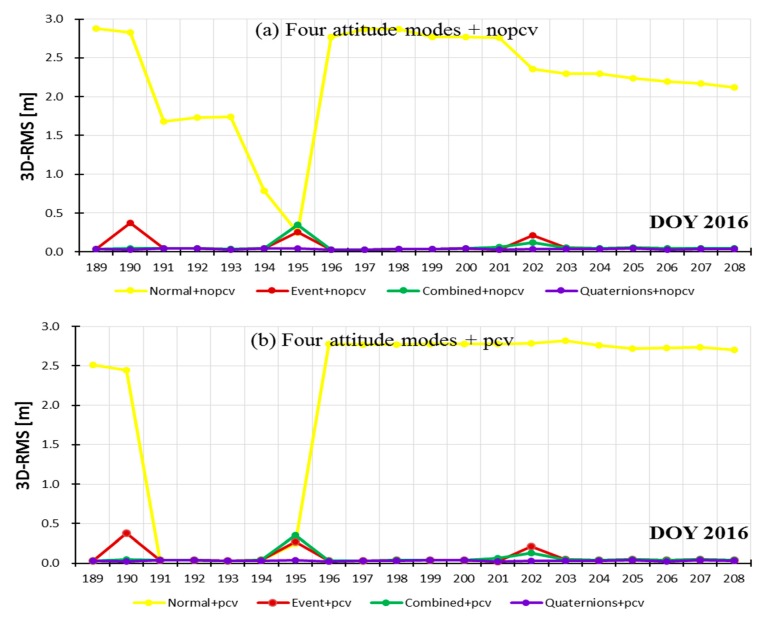
Accuracy comparison of the eight schemes according to with/no PCV.

**Figure 12:**
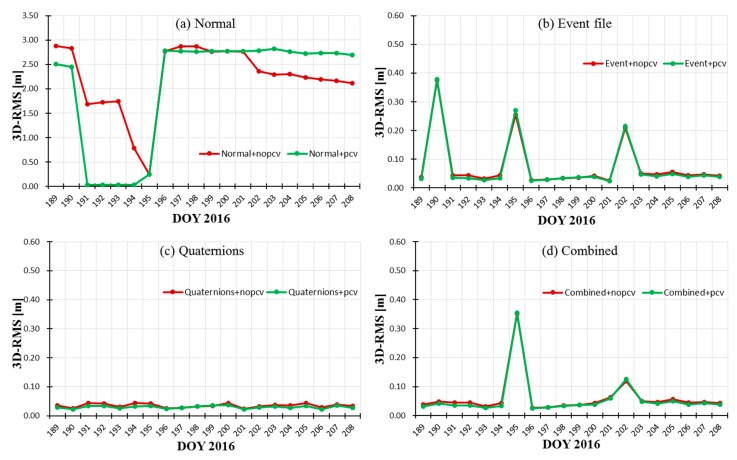
Accuracy comparison of the eight schemes according to different attitude modes.

Figure 11 and Figure 12 show the stability of orbit accuracy (3D-RMS) based on the eight schemes according to with/no PCVs and different attitude modes, respectively. The stability of orbital R-RMS based on eight schemes is similar to those of 3D-RMS; so it will not be shown here. From Figure 11 and Figure 12, we noted that the introduction of PCVs has little influence on the stability of orbit accuracy. Note that Figure 12b–d have the same range of ordinates, which are different from Figure 12a. Among four attitude modes, the stability of orbit accuracy based on quaternions is the best in Figure 12c; the stability of combined attitude is better than that of event file in Figure 12b,d; the normal attitude has the worst results in Figure 12a. The integrated stability of different attitude modes and receiver PCVs on POD are almost consistent with the effects of a single variable.

## 5. Conclusions

The effectiveness of the combined attitude model and receiver PCVs model were investigated in depth. Two strategies about attitude modes and PCV modeling are introduced into Jason-3 POD to integratedly compare their performance. Some conclusions have been drawn:

(1) A combined attitude model based on experimental analysis was designed as the alternative or revision of event file for Jason-3 POD. To validate the feasibility of the combined attitude mode, we analyzed the effects of different attitude modes on PCO estimation, PCO correction and POD. The differences in PCO correction provided a method to define the transition threshold of combined attitude and a validation way for attitude modes. The PCO estimation with combined model are better than those with nominal attitude and slightly worse than those with quaternions. The POD results showed that the orbital accuracy with the combined attitude is slightly more stable than that of event file.
(2) The receiver PCO was estimated in-orbit and the PCVs are modeled by residual approach for Jason-3 satellite. By introducing receiver PCVs map, the orbital accuracy can be improved by millimeter level, in which the mean residuals RMS reduced by 1.9 mm, the 3D-RMS position difference is improved by 5.7 mm, and accuracy of equivalent distance error by SLR validation is improved by 4.1 mm.
(3) The eight schemes were designed to integratedly verify the feasibility of the combined attitude mode and receiver PCVs. The orbital accuracy with receiver PCVs map using nominal attitude, event file, the combined attitude, and quaternions are approximately 197.98 cm, 7.28 cm, 5.70 cm, and 2.97 cm, respectively. The accuracy using the combined attitude is higher than that of event file, which also prove the feasibility of the combined attitude in integrated POD. The integrated effects of attitude modes and receiver PCVs for POD accuracy and stability are almost consistent with the effect of a single variable.

The combined attitude mode provides a revision of event file in the absence of quaternions or inconvenient to use, even in the near-real-time OD. The orbital accuracy can be further improved by integratedly introducing receiver PCVs with all mentioned attitude modes. The optimal POD results indicate that the orbital mean radial RMS are close to 1 cm and the 3D-RMS position difference are within 3 cm, which satisfy the needs of scientific missions for Jason-3 satellite.

## Figures and Tables

**Table 1:** Module/Parameter for Jason-3 POD.

Module/Parameter	Selection/Description
**Force**	
Gravity model	EIGEN-6S4
N-body perturbation	JPL DE405
Solid earth tide	TIDE2000
Ocean tide	FES2004
Solar radiation pressure	ECOM2 model [35]
Atmosphere drag	Pseudo-stochastic pulse
**Data parameter**	
Data type	Code and phase observation of ionosphere-free combination
Data interval	30 s
Cut-off elevation angle	7°
Arc length	24 h
Satellite ephemeris and clock	CODE precise products
Attitude	Attitude model/Quaternions
Receiver antenna PCO/PCV	Direct/Residuals approach
**Estimation parameter**	
Initial state parameters	Six Keplerian osculating elements
Solar radiation pressure	ECOM2 parameters
Receiver clock error	One per epoch as process noise
Pseudo-stochastic pulse	One group per 6 min in the RTN directions
Ambiguities	Float solutions of ionosphere-free combination

**Table 2:** Receiver PCO estimation (z-offset) bias using different attitude modes [unit: mm].

Attitude Mode	Initial Bias	Estimation Bias	Initial Bias	Estimation Bias
Nominal	−24.4	36.2 (148.4%)	−100.2	124.8 (124.6%)
Combined	−24.4	−18.8 (77.0%)	−100.2	−62.8 (62.7%)
Quaternion	−24.4	−3.5 (14.3%)	−100.2	5.1 (5.1%)

**Table 3:** Mean RMS of differences between Jason-3 orbits and CNES orbits [unit: mm].

Our Orbits-CNES	R-RMS	T-RMS	N-RMS	3D-RMS
No PCVs	11.3	27.7	14.5	33.2
PCVs	11.3	21.7	12.7	27.5

**Table 4:** Orbital SLR residuals statistics [unit: mm].

Cut-Off 60°	Mean	STD	RMS	Cut-Off 0°	Mean	STD	RMS
no PCVs	1.1	14.4	14.7	no PCVs	3.3	20.1	21.7
PCVs	−3.7	13.2	14.0	PCVs	−0.8	17.2	17.6

**Table 5:** Accuracy comparison of eight POD schemes [unit: cm].

Scheme	CNES Orbits Validation				SLR Validation		
	R-RMS	T-RMS	N-RMS	3D-RMS	Mean	STD	RMS
1 (Normal + nopcv)	35.89	179.61	78.72	199.36	9.45	71.54	72.62
2 (Event file + nopcv)	2.66	6.65	2.36	7.54	0.23	2.22	2.43
3 (Combined + nopcv)	2.22	5.22	2.16	6.07	0.27	2.21	2.44
4 (Quaternions + nopcv)	1.26	2.83	1.50	3.44	−0.09	1.93	2.11
5 (Normal + pcv)	35.94	178.14	78.55	197.98	8.89	71.58	72.58
6 (Event file + pcv)	2.79	6.38	2.13	7.28	−0.32	2.12	2.29
7 (Combined + pcv)	2.34	4.86	1.85	5.70	−0.27	2.07	2.26
8 (Quaternions + pcv)	1.33	2.38	1.17	2.97	−0.44	1.76	2.01
